# Aneurism of the Superior Palatine Artery

**Published:** 1856-01

**Authors:** 


*Aneurism of the Superior Palatine Artery.* By M. THIERLINCK.

This surgical curiosity was met with in the case of a man, æt. 74. The tumor occupied the roof of the palate, which bled so frequently that the patient was much exhausted. The tumor was soft, elastic, and pulsated synchronously with the heart, alternately expanding and diminishing. Its cause was unknown, and it had lasted for three weeks. The actual cautery was employed, the slough separated in eight days, the hæmorrhage did not recur, and a perfect cure resulted.—*Dublin Hospital Gazette*, from *Gazette Medicale.*

